# Pregnancy induced hypertension and umbilical cord blood DNA methylation in newborns: an epigenome-wide DNA methylation study

**DOI:** 10.1186/s12884-024-06623-8

**Published:** 2024-06-17

**Authors:** Xiaojun Zhu, Peiyue Jiang, Xia Ying, Xueling Tang, Youcai Deng, Xinghong Gao, Xiaofu Yang

**Affiliations:** 1 Department of Obstetrics, Women’s Hospital, Medicine School of Zhejiang University, Hangzhou, 310006 China; 2 Department of Hematology, College of Pharmacy and Laboratory Medicine Science, Army Medical University, Chongqing, 400038 China; 3 School of Basic Medicine, Zunyi Medical University, Zunyi , Guizhou, 563006 China

**Keywords:** Pregnancy induced hypertension, Umbilical cord blood, Whole-genome bisulfite sequencing

## Abstract

**Objectivies:**

Pregnancy induced hypertension (PIH) syndrome is a disease that unique to pregnant women and is associated with elevated risk of offspring cardiovascular diseases (CVDs) and neurodevelopmental disorders in their kids. Previous research on cord blood utilizing the Human Methylation BeadChip or EPIC array revealed that PIH is associated with specific DNA methylation site. Here, we investigate the whole genome DNA methylation landscape of cord blood from newborns of PIH mother.

**Methods:**

Whole-genome bisulfite sequencing (WGBS) was used to examine the changes in whole genome DNA methylation in the umbilical cord blood of three healthy (NC) and four PIH individuals. Using methylKit, we discovered Hypo- and hyper- differentially methylated probes (DMPs) or methylated regions (DMRs) in the PIH patients’ cord blood DNA. Pathway enrichments were assessed using Gene Ontology (GO) and Kyoto Encyclopedia of Genes and Genomes (KEGG) enrichment assays. DMPs or DMRs relevant to the immunological, neurological, and circulatory systems were also employed for enrichment assay, Metascape analysis and PPI network analysis.

**Results:**

520 hyper- and 224 hypo-DMPs, and 374 hyper- and 186 hypo-DMRs between NC and PIH group, respectively. Both DMPs and DMRs have enhanced pathways for cardiovascular, neurological system, and immune system development. Further investigation of DMPs or DMRs related to immunological, neurological, and circulatory system development revealed that TBK1 served as a hub gene for all three developmental pathways.

**Conclusion:**

PIH-associated DMPs or DMRs in umbilical cord blood DNA may play a role in immunological, neurological, and circulatory system development. Abnormal DNA methylation in the immune system may also contribute to the development of CVDs and neurodevelopment disorders.

**Supplementary Information:**

The online version contains supplementary material available at 10.1186/s12884-024-06623-8.

## Introduction

Pregnancy-induced hypertension syndrome (PIH) is a disorder that develops during pregnancy and includes gestational hypertension, preeclampsia, eclampsia, chronic hypertension exacerbated by preeclampsia, and chronic hypertension. The incidence rate of PIH has been approximately 8% over the last decade, with rates of PIH nearly doubling in recent birth cohorts of pregnant women [1, 2]. PIH is a leading cause of morbidity and mortality in pregnant women and infants, resulting in poor fetal growth, intrauterine growth restriction (IUGR) and low birth weight in live individuals [3]. PIH is also a significant risk factor for subsequent illnesses in offspring, such as neurodevelopmental and cardiovascular diseases [4]. Several studies have found that PIH is an independent risk factor for cardiovascular diseases (CVDs) in living individuals [5–8]. Poor maternal cardiovascular health, including PIH, has been linked to poor offspring cardiovascular health, including body-mass index, blood pressure, total cholesterol level, and glucose level, in a recent multinational cohort study involving 2302 mother-infant dyads [9]. Additionally, a prospective study discovered a favorable correlation between the blood pressure of the mother and the blood pressure of the kids during infancy and adolescence [10]. There have been reports of increased blood pressure [11], thicker blood vessel walls, and decreased left ventricular end-diastolic volume in children born to preeclamptic mothers [12]. Thus, it's critical to comprehend the mechanisms underlying lower cardiovascular health for both mother and offspring is important.

DNA methylation patterns provide a rich source of novel biomarkers and insights into various disease etiologies. Epigenetic changes in the placenta during preeclampsia affect fetal development and chronic diseases [13]. Umbilical cord blood is a popular choice for studying the genetic, epigenetic, and environmental mechanisms of the developmental origin of health and disease [14]. For instance, DNA methylation from umbilical cord blood may serve as biomarkers to forecast the likelihood of both poor neurodevelopment and unfavorable early life experiences [15]. Using the Illumina Human Methylation 450 BeadChip, two previous studies investigated alterations in DNA methylation in cord blood from PIH [16, 17]. Numerous deferentially methylated sites related to developmental, embryonic, or neurological processes and connected with PIH are revealed by these investigations [16]. According to a recent study using cord blood and the Illumina Human Methylation EPIC array, the identified PIH-associated methylated CpGs were found to be enriched in the birth weight-related differentially methylated CpGs [18]. Nevertheless, nothing is known about the precise patterns of genome-wide DNA methylation in PIH cord blood.

In this study, we determined whole genome DNA methylation in the cord blood from three healthy donors (normal control, NC) and four PIH patients using whole-genome bisulfite sequencing (WGBS). According to Gene Ontology (GO) and Kyoto Encyclopedia of Genes and Genomes (KEGG) enrichment assays, the development of the cardiovascular, nervous, and immune systems in offspring was found to be associated with the differentially methylated probes (DMPs) and differentially methylated regions (DMRs) between the NC and PIH groups. It was also revealed that TBK1 was the hub gene with DNA methylation changes among all these three developmental pathways.

## Material and methods

### Sample collection

Umbilical cord blood from three NC and four PIH neonates was collected using heparin-containing tubes after delivery of the baby but before delivery of the placenta by trained staff at Zhejiang University's Women's Hospital from December 2022 to April 2023 [16, 18]. This study was approved by the Ethics Committee of Women's Hospital of Zhejiang University (No. IRB-20220390-R) and carried out in accordance with the World Medical Association (Declaration of Helsinki) Code of Ethics for human experimentation. Every patient provided informed consent. The clinical features of PIH patients and NC neonates are shown in Table 1. For PIH patients, including one gestational hypertension, two mild preeclampsia, and one severe preeclampsia. Gestational hypertension was defined in pregnant women who were previously normotensive and had at least two occasions four or more hours from increase in systolic blood pressure (SBP) of at least 30 mmHg and/or a rise from baseline diastolic blood pressure (DBP) of at least 15 mmHg, or SBP ≥ 140 mmHg and/or DBP ≥ 90 mmHg. Pre-eclampsia was defined in pregnant women who were previously normotensive and had at least two occasions six or more hours apart a rise in SBP of at least 30 mmHg and/or a rise from baseline DBP of at least 15 mmHg, or SBP ≥ 140 mmHg and/or DBP ≥ 90 mmHg. Women with pre-existing hypertension or other medical conditions (e.g., kidney disease, diabetes, twin pregnancies, or foetal abnormalities) associated with pre-eclampsia were excluded.

**Table 1:** Maternal and newborn characteristics of participants

Sample symbol	Mother Age	Gestational age (weeks)	PIH characteristics	Prepregnancy BMI, kg/m^2^	Weight of New born (g)	Baby’s sex	Notes
NC_1	33	38 + W	Healthy	21	3650	Female	Non Prepregnancy diabetes and Non pregnancy diabetes
NC_3	36	38 + W	Healthy	19.87	4200	Male	Non Prepregnancy diabetes and Non pregnancy diabetes
NC_12	38	38 + W	Healthy	18.4	4030	Male	Non Prepregnancy diabetes and Non pregnancy diabetes
HIP-1	37	38 W	Severe Preeclampsia	22.7	3510	Female	Non Prepregnancy diabetes and Non pregnancy diabetes
HIP-2	41	38 + W	Severe Gestational Hypertension that progresses to Mild Preeclampsia	27	3360	Male	Non Prepregnancy diabetes and Non pregnancy diabetes
HIP-3	45	37 + W	Severe Gestational Hypertension that progresses to Mild Preeclampsia. Placental lesions: placenta size 19*14*1.5 cm, weight 230 g (< 3 percentile), advanced placental tissue, local interplacental hematoma, local fibrin deposition around the villi with increased synaptic nodules	21	2240	Female	Non Prepregnancy diabetes and Non pregnancy diabetes
HIP-4	28	38 + W	Gestational Hypertension. Placental lesions: placenta size 18*15*2.0 cm, weight 475 g (< 2 percentile), the development of placental villi conforms to the gestational age, local fibrin deposition around the villi, and local villi degeneration with increased syncytial nodules	23	2720	Female	Non Prepregnancy diabetes and Non pregnancy diabetes

### WGBS, raw data processing and read alignment

After sampling, cord blood samples were placed on damp ice, and then sent right away to Hangzhou LC-BIO Bio-tech Ltd (Hangzhou, China). Genomic DNA (gDNA) was isolated using a Gentra Puregene Blood Kit (Qiagen) and stored at -80 °C until used according to the manufacturer’s instructions. A Qubit4 Fluorometer (Life Technologies, USA) was used to quantify gDNAs. DNA library construction and sequencing was performed using Acegen Bisulfite-Seq Library Prep Kit (Acegen, Cat. No. AG0311) following the protocols prescribed by LC-BIO Bio-tech Ltd [19]. Briefly, 1 μg of genomic DNA spiked with 1 ng unmethylated Lambda DNA was fragmented by sonication to an average size of roughly 200–500 bp, end-repaired, 5'-phosphorylated, 3'-dA-tailed and ligated with 5-methylcytosine-modified adapters. Then, the DNA was amplified with 10 cycles of PCR using Illumina 8-bp dual-index primers after bisulfite treatment. The constructed WGBS libraries were then analyzed using the Agilent 2100 Bioanalyzer and finally sequenced using the Illumina HiSeq 150 × 2 paired-end sequencing protocol at an average depth of 30 × .

The raw data were converted to FASTQ format using the Illumina package bcl2fastq v2.15.0, and the resulting reads were used as input for further bioinformatics analyses. Raw read statistics are shown in Supplementary Table S1. The** s**equencing data were aligned to human reference genome hg38 using Bsmap software using software default alignment parameters [20]. The SAM files obtained for each sample were converted to BAM files using Samtools software [21]. Subsequently, the obtained BAM files (Supplementary Table S2) was sorted and deduplicated using Sambamba software [22]. MethylDackel (https://github.com/dpryan79/MethylDackel) was utilized to extract the methylation information of each sample, based on the human reference genome hg38 used for the alignment.

### Methylation data statistics and annotationof DMPs and DMRs

Since DNA methylation can occur in CpG, CHG and CHH contexts and methylKit can be used to provide methylation information for all of these contexts from SAM files, we next utilized methylKit in the R package to estimate the percentage of methylation, read coverage distributions, and filter bases with very high read coverage that caused by PCR duplication bias (filterByCoverage, lo.count = 10, hi.perc = 99.9), perform batch effect correction, and conduct principal component analysis (PCA) [23].

Using methylKit, DMPs were also identified by comparing methylated CpG sites that were smaller or more than the NC group (|meth.diff |> 0 & *p*value < 0.01, logistic regression) [23]. DMRs were defined using a sliding window approach with a window size of 1000 bp and a step size of 1000 bp, using the “tileMethylCounts function” (meth.diff |> 0 & *p*value < 0.01, logistic regression) [23].

### Annotation and functional analysis of DMPs and DMRs

To assess the biological impact of the DMPs and DMRs obtained above, we performed annotation using the GenomicRanges package in the R language. This annotation involved identifying differentially methylated genes as well as determining the nearest transcription start site (TSS) and gene components [24]. For the enrichment analysis of genes associated with DMPs and DMRs, the ClusterProfiler R package was used for GO biological process and KEGG pathway analysis [25].

Given the close relationship between PIH and immunity and inflammation, we downloaded and sorted the list of immune-related genes (IRGs) from the ImmPort database(https://immport.niaid.nih.gov) database [26]. We then intersected this list with the differentially methylated genes associated with DMPs or DMRs to obtain the differentially methylated immune genes. From these genes, we selected the key differentially methylated immune genes, which were further analyzed for their functions using the Metascape database [27]. Similar analysis were conducted for DMPs and DMRs related to the nervous and cardiovascular systems.

## Results

### Analysis of differentially methylated probes (DMPs) and regions (DMRs)

**Fig. 1:**
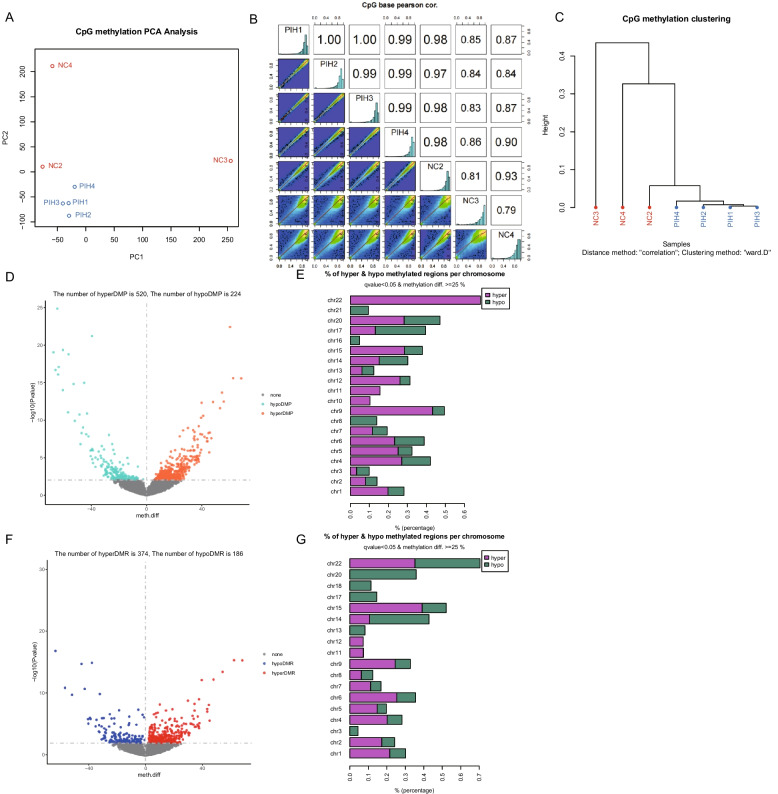
Analysis of differentially methylated probes (DMPs) and regions (DMRs). PCA correlation analysis (**A**), the correlation between samples in NC group and in PIN group (**B**) and clustering (**C**) of samples between health donors (NC) and HIP group by PCA analysis, respectively. **D**, **F**. Differential methylation probe (DMP) (**D**) and region differential methylation (DMR) (**F**) between NC and HIP groups, respectively. **E**, **G**. Percentage of hyper- or hypo-DMP or DMR in each chromosome between NC and HIP groups

WGBS was performed on 7 individuals, including 3 healthy donors and 4 individuals with PIH. Raw sequenced reads were converted to FASTQ format, and at least 95% of reads had a Phred score of > 20. After removing duplicate and misaligned reads, over 50% of these reads were retained. Following the correction of batch effects, the correlation between samples in the same group was significantly higher than the correlation between groups (Fig. 1A and B). Cluster analysis further confirmed the results of the correlation analysis (Fig. 1C). The DMP analysis revealed 520 hyper- and 224 hypo-DMPs, while the DMR analysis revealed 374 hyper- and 186 hypo-DMRs between the NC and PIH groups, respectively (loci with a methylation change > 1.5-fold and a q-value < 0.05; Supplementary Data 1 and 2) (Fig. 1D,F).

We also analyzed the distribution of DMPs or DMRs in each chromosome and found that chromosome 22 exhibited more methylation changes than other chromosomes between the NC and PIH groups (Fig. 1E,G). Chromosome 22 is the shortest human chromosome and carries approximately 52, 000 kb of DNA. Diseases associated with chromosome 22 include chronic myeloid leukaemia, meningioma and nervous system malignancies, and others [28]. Further analysis revealed that methylated genes on chromosome 22 are primarily involved in congenital heart disease, immunodeficiency, schizophrenia, mental retardation, and congenital defect, suggesting that PIH syndrome may lead to disorders of cardiovascular, immune, and nervous system development. Furthermore, within the top 20 hyper-DMR and hypo-DMR-related genes (Table S3), several genes are associated with severe diseases. For example, FANCL has been associated with Fanconi anemia and premature ovarian insufficiency, and EPM2A has been linked to Lafora disease [29–31].

### GO and KEGG analysis of DMRs between NC and PIH group

**Fig. 2:**
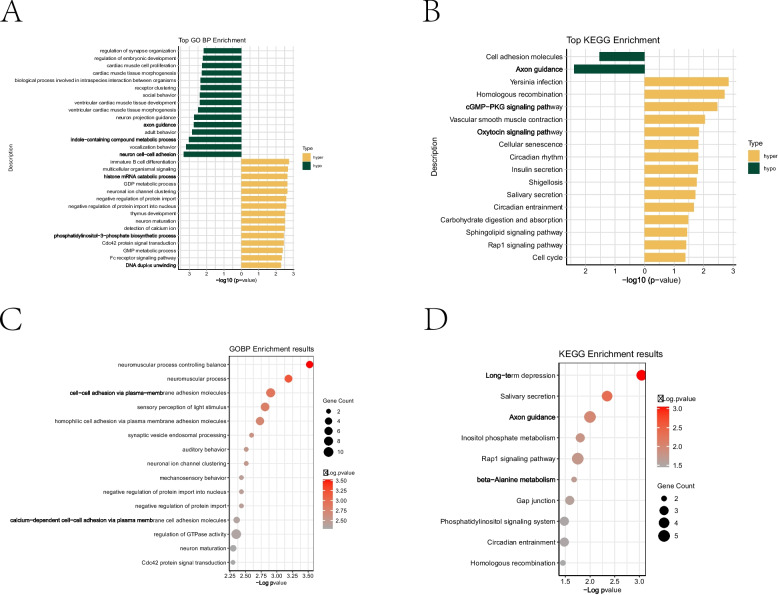
GO and KEGG pathway enrichment analysis of genes with DMPs and DMRs between NC and PIHgroup. **A**-**D** GO biological process (**A**, **C**) and KEGG pathway analysis (**B**, **D**) of DMPs (**A**,**B**) and DMRs (**C**,**D**) between NC and PIH groups

To further investigate the potential impact of the genome-wide DNA methylation changes in umbilical cord blood from PIH, we conducted GO biological process and KEGG pathway enrichment analysis for both DMPs and DMRs. The GO biological processes analysis of DMPs revealed their involvement in various processes, including the regulation of embryonic development, nervous system development (adult behavior, vocalization behavior, auditory behavior, mechanosensory behavior, etc.), and the immune-related biological processes (cell–cell adhesion via plasma-membrane adhesion molecules, homophilic cell adhesion via plasma membrane adhesion molecules, immature B cell differentiation, etc.) (Fig. 2A). Similarly, the KEGG pathway analysis indicated that DMPs were primarily involved in embryonic development (Homologous recombination, Circadian entrainment, Circadian rhythm, etc.), cardiovascular development (Vascular smooth muscle contration, arrhythmogenic right ventricular cardiomyopthy, hypertrophic cardiomyopathy, dilated cariomyopathy) and immune-related pathways (Yersinia infection, Rap1 signaling pathway, Cell adhesion molecules, Cellular senescence, etc.). Notably, changes in Vascular smooth muscle contraction and Oxytocin signaling pathway may be associated with pregnancy termination due to eclampsia (Fig. 2B).

The GO biological process and KEGG analysis of DMRs yielded similar findings to DMPs. The GO biological process analyses indicated the involvement of DMRs in nervous system development (neuromuscular process controlling balance, sensory perception of light stimulus, synaptic vesicle endosomal processing, auditory behavior, etc.) and immune-related biological processes (cell–cell adhesion via plasma-membrane adhesion molecules, homophilic cell adhesion via plasma membrane adhesion molecules, etc.) (Fig. 2C). The KEGG pathway analysis also revealed that DMRs are primarily involved in nervous system development (long-term depression, Axon guidance, etc.), immune-related pathway (Inositol phosphate metabolism, Rap1 signaling pathway, Gap junction, Homologous recombination, etc.) (Fig. 2D).

Overall, the above results suggest that PIH induces significant DNA methylation changes in genes related to nervous system development, cardiovascular development and immune-related pathways (Table 2). These changes play a crucial role in the development of CVDs and neurodevelopmental disorders in offspring.

**Table 2:** Gene list for three types gene DMR

	**chr**	**start**	**end**	**strand**	*p* value	**meth.diff**	**type**	**GeneSymbol**
**Immune genes**	chr1	107,606,001	107,607,000	*	0.0048948325146922	16.0714285714286	hyperDMR	VAV3
	chr1	107,703,001	107,704,000	*	0.00719235069720498	24.1031941031941	hyperDMR	VAV3
	chr1	216,958,001	216,959,000	*	7.49589283386365e-05	22.7421271538919	hyperDMR	ESRRG
	chr5	78,064,001	78,065,000	*	0.00462013086508546	18.5263562908811	hyperDMR	AP3B1
	chr6	152,054,001	152,055,000	*	0.00639057018128456	7.98031541383677	hyperDMR	ESR1
	chr8	32,353,001	32,354,000	*	1.8724989796978e-05	-30.6280193236715	hypoDMR	NRG1
	chr12	64,461,001	64,462,000	*	0.000764167033465426	9.92616899097621	hyperDMR	TBK1
	chr14	64,312,001	64,313,000	*	0.0056585533825182	-5.95238095238095	hypoDMR	ESR2
**Nervous system genes**	chr4	93,692,001	93,693,000	*	0.00575145963907569	-5.53359683794467	hypoDMR	GRID2
	chr4	102,379,001	102,380,000	*	0.000291764265158561	-13.5690594645819	hypoDMR	SLC39A8
	chr8	32,353,001	32,354,000	*	1.8724989796978e-05	-30.6280193236715	hypoDMR	NRG1
	chr8	52,677,001	52,678,000	*	0.00560134362426618	-8.98408725826921	hypoDMR	RB1CC1
	chr9	130,735,001	130,736,000	*	0.00561022896122505	9.375	hyperDMR	ABL1
	chr9	32,561,001	32,562,000	*	0.00280905785086238	10.2040816326531	hyperDMR	SMIM27
	chr10	51,142,001	51,143,000	*	0.00361936387422146	18.1352459016393	hyperDMR	PRKG1
	chr11	106,814,001	106,815,000	*	0.00872959670196106	-5.52995391705069	hypoDMR	GUCY1A2
	chr12	64,461,001	64,462,000	*	0.000764167033465426	9.92616899097621	hyperDMR	TBK1
	chr20	8,583,001	8,584,000	*	0.00275471730244881	8.33333333333334	hyperDMR	PLCB1
**Circulatory system genes**	Chr1	107,606,001	107,607,000	*	0.004894833	16.07142857	hyperDMR	VAV3
	Chr6	129,133,001	129,134,000	*	2.51E-03	-30.58312655	hypoDMR	LAMA2
	Chr9	130,735,001	130,736,000	*	0.005610229	9.375	hyperDMR	ABL1
	Chr9	32,561,001	32,562,000	*	0.002809058	10.20408163	hyperDMR	NDUFB6
	Chr10	51,142,001	51,143,000	*	0.003619364	18.1352459	hyperDMR	PRKG1
	Chr11	106,814,001	106,815,000	*	0.008729597	-5.529953917	hypoDMR	GUCY1A2
	Chr12	64,461,001	64,462,000	*	0.000764167	9.926168991	hyperDMR	TBK1
	Chr12	1,901,001	1,902,000	*	0.003076533	10.46245421	hyperDMR	CACNA2D4
	Chr20	8,583,001	8,584,000	*	0.002754717	8.333333333	hyperDMR	PLCB1

### Differentially methylated immune response-related genes (IRGs) in PIH

**Fig. 3:**
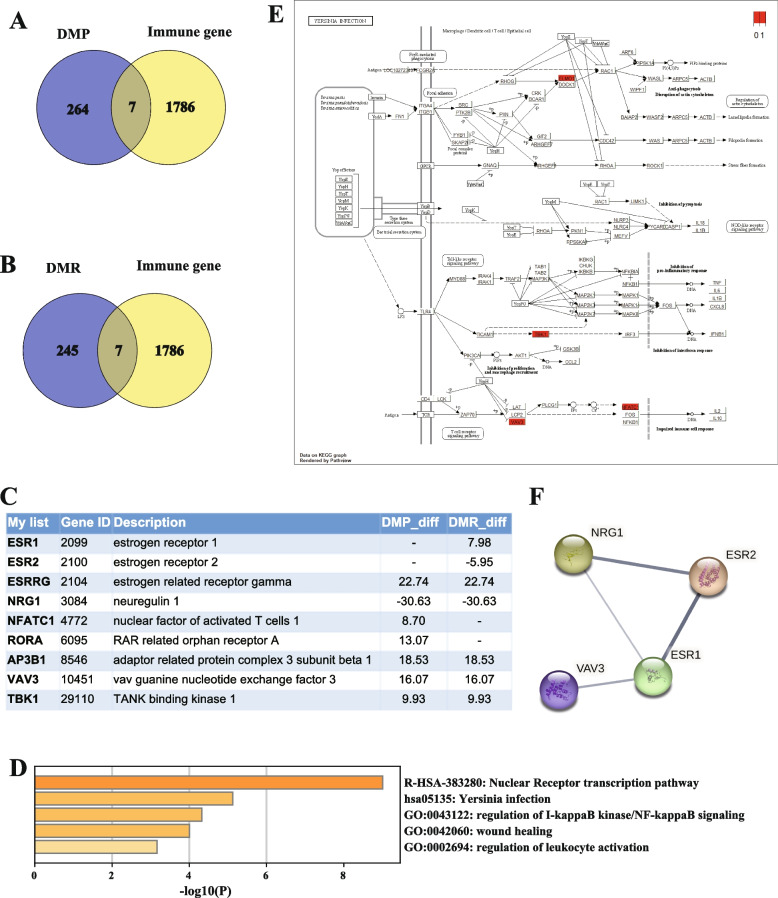
Differentially methylated immune response-related genes (IRGs) in PIH. **A** Immune-related genes in the DMPs. **B** Immune-related genes in the DMRs. **C** List of immune-related genes list in the DMPs and DMRs. **D** Metascape analysis of the immune-related genes in the DMPs and DMRs. **E** KEGG map of Yersinia infection. **F** PPI network analysis

Since our above findings demonstrated significant DNA methylation changes in immune-related pathways due to PIH, we proceeded to examine the genes associated with DMPs or DMRs in these pathways. After crossing DMPs or DMRs with genes in the immune-related pathways, we identified 7 genes with DMPs between the PIH and NC groups: *AP3B1, ESRRG, NFATC1, NRG1, RORA, TBK1, VAV3* (Fig. 3A). Additionally, we found 7 genes with DMRs between the PIH and NC groups: *AP3B1, ESR1, ESR2, ESRRG, NRG1, TBK1, VAV3* (Fig. 3B). Therefore, our subsequent focus was on these 9 genes (Fig. 3C), and the IGV analysis of these genes is presented in Figure S1. Metascape analysis further indicated that these 9 genes were mainly enriched in the Nuclear receptor transcription pathway, Yersinia infection, regulation of *I-kappaB kinase/NF-kappaB* signaling, and regulation of leukocyte activation (Fig. 3D). From the KEGG map of Yersinia infection, it is evident that significant methylation changes in *VAV3, TBK1,* and *NFATC1* subsequently influence the downstream interferon response and immune cell response (Fig. 3E). PPI network analysis showed that *NRG1, ESR2, ESR1 and VAV3* interacted with each other in the network of IRGs (Fig. 3F).

### Differentially methylated nervous system development in PIH

**Fig. 4:**
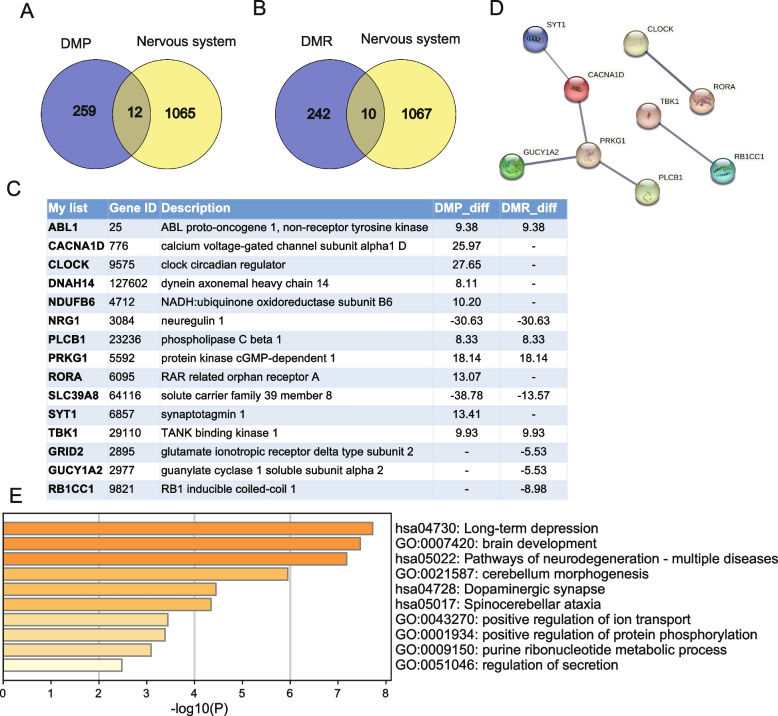
Differentially methylated genes related nervous system development in PIH. **A** Nervous system development-related genes in the DMPs. **B** Genes related to nervous system development in the DMRs. **C** List of immune-related genes list in the DMPs and DMRs. **D** PPI network analysis. **E** Metascape analysis of nervous system development-related genes in the DMPs and DMRs

Next, we examined the intersection of DMPs or DMRs with genes related to nervous system development. We identified 12 genes with DMPs in signaling pathways related to the nervous system: *ABL1, CACNA1D, CLOCK, DNAH14, NDUFB6, NRG1, PLCB1, PRKG1, RORA, SLC39A8, SYT1,* and *TBK1* (Fig. 4A). Furthermore, we found 10 genes with DMRs in pathways related to nervous system: *ABL1, GRID2, GUCY1A2, NRG1, PLCB1, PRKG1, RB1CC1, SLC39A8, SMIM27,* and *TBK1* (Fig. 4B). Metascape analysis revealed that these 15 genes were mainly enriched in Long-term depression, Brain development, Pathways of neurodegeneration-multiple diseases, Cerebellum morphogenesis, opaminergic synapse, and et al. (Fig. 4C and E). The IGV analysis of these genes is shown in Figure S2. PPI network analysis revealed interactions among *PRKG1, GUCY1A2, PLCB1, CACNA1D* and *SYT1* (Fig. 4D).

### Differentially methylated circulatory system development in PIH

**Fig. 5:**
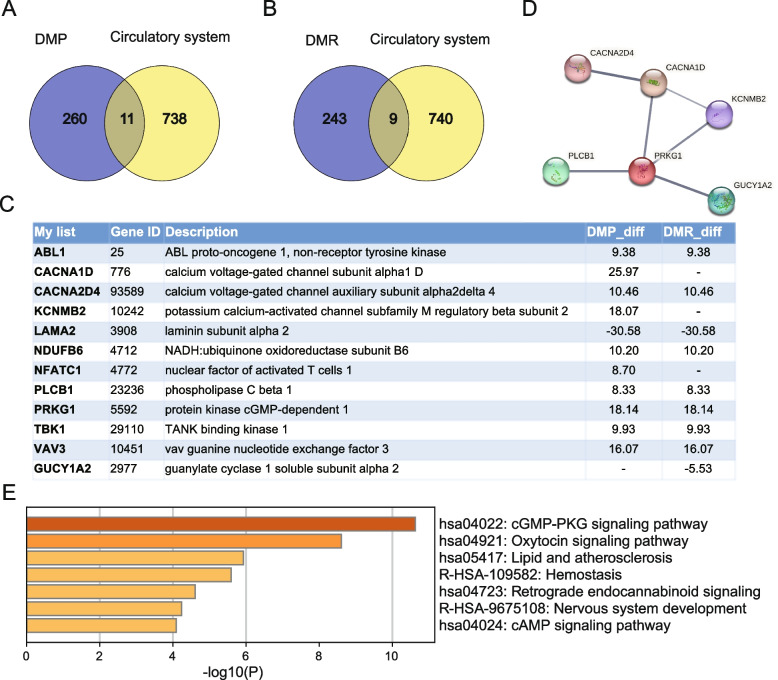
Differentially methylated genes associated with circulatory system development in PIH. **A** Genes in the DMPs associated with circulatory system development in the DMPs. **B** Genes in the DMPs associated with circulatory system development in the DMRs. **C** List of immune-related genes list in the DMPs and DMRs. **D** PPI network analysis. **E** Metascape analysis of genes associated with circulatory system development in the DMPs and DMRs

Finally, we focus on genes related to circulatory system development that exhibited DMPs or DMRs. We identified 11 genes with DMPs: *ABL1, CACNA1D, CACNA2D4, KCNMB2, LAMA2, NDUFB6, NFATC1, PLCB1, PRKG1, TBK1,* and *VAV3* (Fig. 5A). Additionally, we found 9 genes with DMRs: *ABL1, CACNA2D4, GUCY1A2, LAMA2, NDUFB6, PLCB1, PRKG1, TBK1,* and *VAV3* (Fig. 5B). The IGV peak of these 12 genes were shown in Figure S3. Metascape analysis revealed that these genes with DMPs or DMRs related to circulatory system development were mainly enriched in cGMP-PKG signaling pathway, Oxytocin signaling pathway, Lipid and atherosclerosis, Retrograde endocannabinoid signaling, and cAMP signaling pathway (Fig. 5C and E). PPI network analysis also showed interactions among *PRKG1, CACNA1D, CACNA2D4, KCNMB2, PLCB1,* and *GUCY1A2* (Fig. 5D).

## Discussion

PIH has significant physical effects on both the mother and child. For instance, it can lead to various complications for the mother, including high blood pressure, arterial thickening, and myocardial infarction. In addition, it can have negative effects on the offspring, such as autism spectrum disorder (ASD), attention-deficit/hyperactivity disorder (ADHD), depression [32, 33], as well as increased left ventricular wall thickness or decreased left ventricular end-diastolic volume [34, 35]. Previous studies utilizing the Illumina HumanMethylation450 BeadChip or Illumina Human Methylation EPIC array have demonstrated that DNA methylation changes in the cord blood from newborns affected by PIH are associated with genes involved in developmental and neurological pathways [16–18]. Consistently, our comprehensive analysis of the DNA methylome revealed that DNA methylation in cord blood of PIH neonates enriched in genes related to CVDs and neurodevelopmental disorders. Furthermore, our study also exhibited enrichment of DNA methylation in IRGs, suggesting a potential immunological abnormality in the offspring of mothers with PIH, warrants epidemiological and evidence-based investigation.

Several genes with DMPs or DMRs in cord blood from PIH-affected individuals, as identified in our study, have already been associated with cardiovascular and neuronal development. The ABL1 gene, which encodes a protein kinase involved in cellular response to stress, differentiation, and adhesion, plays an important role in cardiac development, and a gain-of-function mutation in ABL1 leads to congenital heart defects [36]. A previous study also showed that ABL1 methylation is upregulated in preeclampsia [37]. CACNA1D is involved in the regulation of blood pressure [38], and alterations in CACNA1D methylation have been observed in neonates exposed to maternal hyperglycemia [39]. The CLOCK gene is a critical gene involved in the regulation of the circadian clock and clock-controlled genes. Some evidence suggests that preeclampsia (PE) is controlled through the alteration of the circadian rhythm homeostasis. Previous studies have found DNA methylation changes in circadian clock and clock-controlled genes in the placenta and neonatal tissues of early-onset and late-onset preeclampsia (EOPE and LOPE), and consistent with our findings, differential DNA methylation of the CLOCK gene was observed in umbilical cord leukocytes (UCL) of EOPE compared to uncomplicated controls [40]. NFATC1 coordinates the development of valve endocardial cells necessary for heart valve formation, and missense mutations in NFATC1 are associated with atrioventricular septal defect [41, 42]. VAV3, a regulator of vascular smooth muscle cell proliferation and migration involved in vascular wall remodeling, has been associated with cardiovascular risk factors and an increased risk of developing hypertension [43, 44]. FANCL was found to be associated with biallelic loss-of-function mutations in FANCL result in systemic autosomal recessive microangiopathy and focal neurological deficits, including hemiparesis, aphasia, and seizures occurring in adolescence [45]. Homozygous EPM2A mutation (NM_005670.3; c.838 T > G; pTrp280Gly) leads to early-onset parkinsonism and severe neurocognitive decline [46], whereas in-frame deletion of EPM2A slows neurological decline in Lafora disease [47]. NRG1 is required for neuronal development and is important for neurotransmission and synaptic plasticity [48]. SLC39A8 polymorphism has been linked to neurodevelopment and schizophrenia in children [49, 50], and hypomethylated SLC39A8 has been shown to be overexpressed in preeclampsia [37]. CACNA1D has been identified as an autism risk gene [51]. Recently, DNAH14 and SYT1 variants have been implicated in neurodevelopmental disorders [52, 53]. Hence, abnormal methylation in these genes may mediate the effects of PIH on cardiovascular and neuronal development in offspring.

In addition to the cardiovascular and nervous systems, our study also found increased methylation in genes related to immunity. A previous study indicated that infants of preeclamptic mothers have a lower proportion of FoxP3^+^ regulatory T cells, a lower CD4/CD8^+^ T cell ratio, and a higher percentage of CD56^lo^CD16^+^ effector NK cells compared to CD56^hi^CD16^−^ regulatory NK cells [54]. Another study associated preeclampsia to fetal thymic hypoplasia, characterized by a 38% decrease in thymic volume and a 12.2% decrease in diameter [55]. These immune system alterations in the offspring of preeclamptic mothers may be associated with epigenetic changes [56]. In our study, several IRGs with DMPs or DMRs play important roles in immune system development and function. NFATC1 has been shown to regulate both B and T cell development [57]. The synergistic defect of AP3B1 with UNC13D can lead to hemophagocytic lymphohistiocytosis [58]. Dysregulation of the immune system plays a pivotal role in the development of CVDs and neurodevelopmental disorders. Thus, our results provide further evidence of a potential immune abnormality in offspring of mothers with PIH, which may contribute to the development of CVDs and neurodevelopment disorders in later life.

Notably, several genes with DMPs or DMRs are enriched in pathways related to all the aforementioned systems (cardiovascular, immune, and nervous systems), such as TBK1, NFATC1 and CACNA1D. In particular, TBK1 is involved in the development and function of the cardiovascular, immune and nervous systems [59–61]. TBK1 plays a critical role in vascularization and the differentiation of follicular helper T cells in the germinal center [59, 60]. Several studies have also demonstrated that TBK1 mutations are associated with the development of amyotrophic lateral sclerosis, an adult-onset neurodegenerative disorder affecting motor neurons [61].

## Conclusion

In summary, the induction of PIH leads to DMPs and DMRs in cord blood, resulting in abnormal DNA methylation in critical genes related to nervous system development, cardiovascular development and immune-related pathways. These alterations may contribute to corresponding disorders in the offspring. Moreover, the methylation patterns identified in our study may serve as potential biomarkers of cardiovascular, immune, and neurological diseases in children and adolescents, particularly in the context of PIH neonates.

However, there are limitations of our study that should be acknowledged. These include the relatively small sample size, the absence of methylation analysis in CpG islands, promoters, or enhancers, and a need for functional validation of gene methylation. These limitations highlight the need for further investigations in this field.

### Supplementary Information

Supplementary Material 1.

Supplementary Material 2.

Supplementary Material 3.

Supplementary Material 4.

## Data Availability

The data that support the findings of this study are available on NCBI data base (https://immport.niaid.nih.gov, BioProject: PRJNA1030145). If you would like to see a description of these 7 samples, click on links as follows: https://trace.ncbi.nlm.nih.gov/Traces/?view=run_browser&acc=SRR26446886&display=data-access https://trace.ncbi.nlm.nih.gov/Traces/?view=run_browser&acc=SRR26446887&display=data-access https://trace.ncbi.nlm.nih.gov/Traces/?view=run_browser&acc=SRR26446888&display=data-access https://trace.ncbi.nlm.nih.gov/Traces/?view=run_browser&acc=SRR26446889&display=data-access https://trace.ncbi.nlm.nih.gov/Traces/?view=run_browser&acc=SRR26446890&display=data-access https://trace.ncbi.nlm.nih.gov/Traces/?view=run_browser&acc=SRR26446891&display=data-access https://trace.ncbi.nlm.nih.gov/Traces/?view=run_browser&acc=SRR26446892&display=data-access If you want to check the details of the data, please download them as follows: https://sra-pub-run-odp.s3.amazonaws.com/sra/SRR26446887/SRR26446887 https://sra-pub-run-odp.s3.amazonaws.com/sra/SRR26446886/SRR26446886 https://sra-pub-run-odp.s3.amazonaws.com/sra/SRR26446888/SRR26446888 https://sra-pub-run-odp.s3.amazonaws.com/sra/SRR26446889/SRR26446889 https://sra-pub-run-odp.s3.amazonaws.com/sra/SRR26446890/SRR26446890 https://sra-pub-run-odp.s3.amazonaws.com/sra/SRR26446891/SRR26446891 https://sra-pub-run-odp.s3.amazonaws.com/sra/SRR26446892/SRR26446892
