# Supplementary material for: Pregnancy induced hypertension and umbilical cord blood DNA methylation in newborns: an epigenome-wide DNA methylation study
Source: BMC Pregnancy Childbirth. 2024 Jun 17;24:433. doi: 10.1186/s12884-024-06623-8 (PMC11181590; doi:10.1186/s12884-024-06623-8)
Supplement: Supplementary file 1 — Supplementary Material 1. [file 12884_2024_6623_MOESM1_ESM.pdf]

Supplemenatal Materials

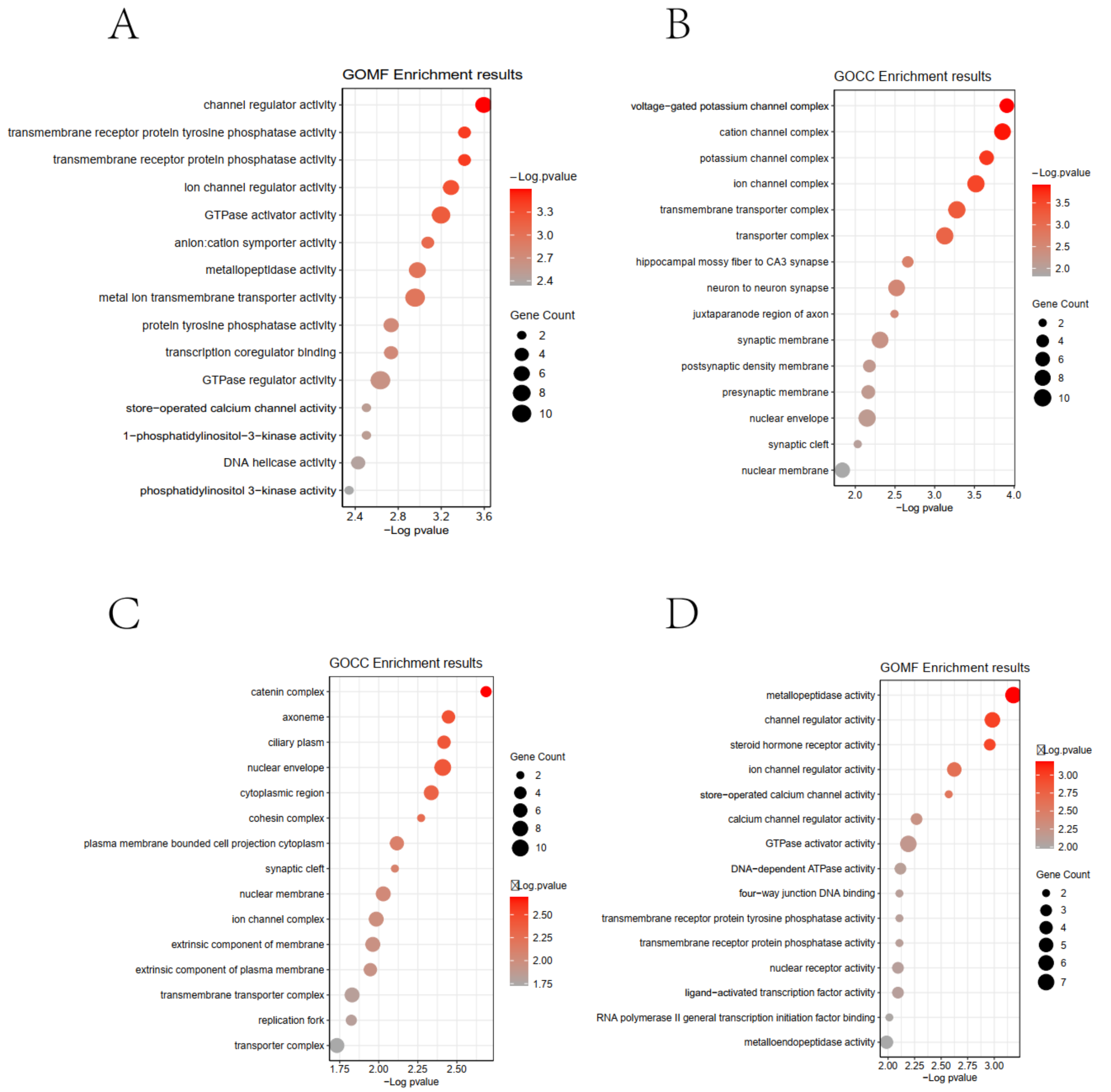

Fig S1. GOCC and GOMP enrichment analysis of genes with DMPs and DMRs between NC and PIHgroup. A-D GO cell composition (A, C) and GO molecular function analysis (B, D) of DMPs (A,B) and DMRs (C,D) between NC and PIH groups.

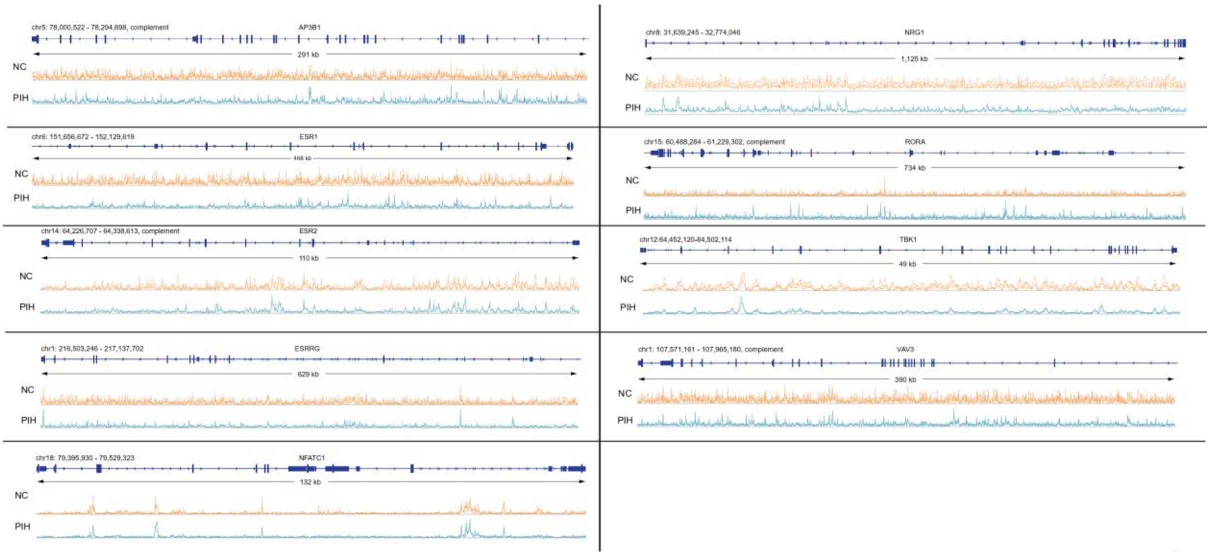

Fig S2. The IGV analysis of immune response-related genes in the DMPs and DMRs.

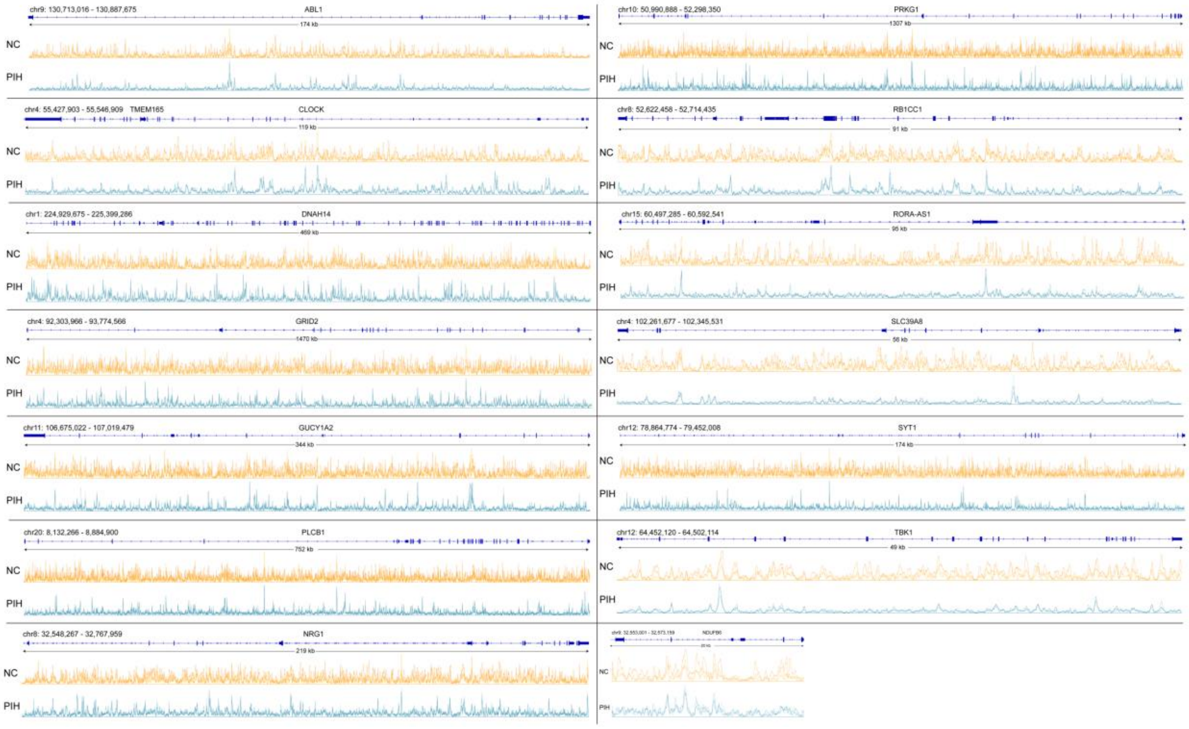

Fig S3. The IGV analysis of nervous system development-related genes in the DMPs and DMRs.

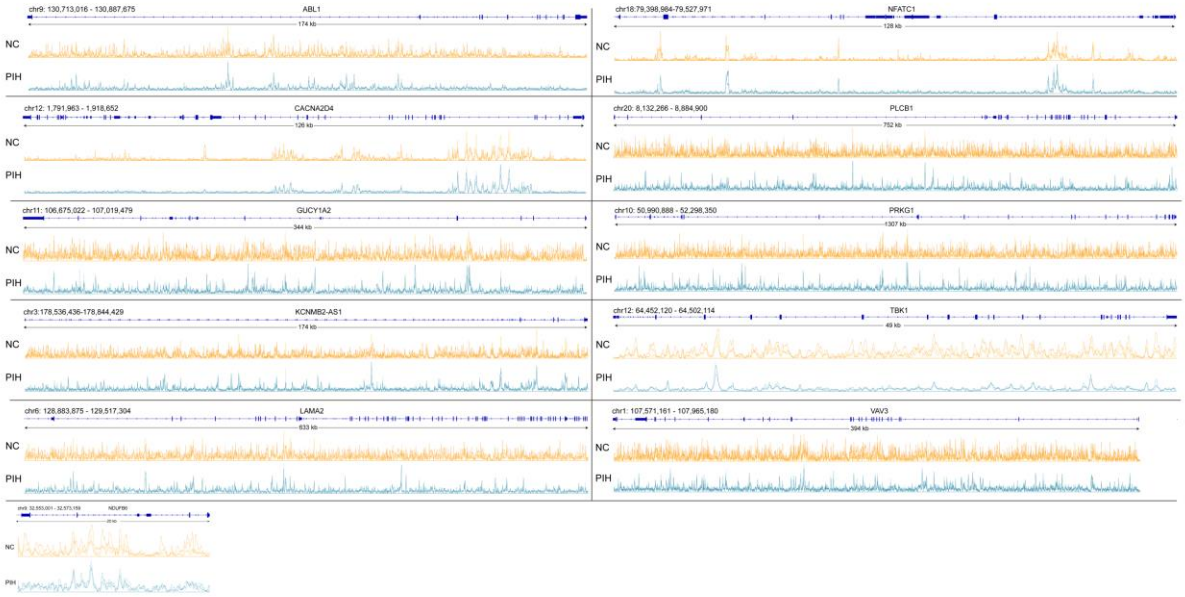

Fig S4. The IGV analysis of **circulatory system development**-related genes in the DMPs and DMRs.
