# Supplementary material for: Pregnancy induced hypertension and umbilical cord blood DNA methylation in newborns: an epigenome-wide DNA methylation study
Source: BMC Pregnancy Childbirth. 2024 Jun 17;24:433. doi: 10.1186/s12884-024-06623-8 (PMC11181590; doi:10.1186/s12884-024-06623-8)
Supplement: Supplementary file 2 — Supplementary Material 2. [file 12884_2024_6623_MOESM2_ESM.docx]

**Table S1.** **The bsmap software compares the methylation sequencing data of each sample with the reference genome hg19.**

| Sample | Total read pairs | Aligned pairs | Unique pairs | Non-unique pairs | Unpaired read #1 | Unique reads | Non-unique reads | Unpaired read #2 | Unique reads | Non-unique reads |
| --- | --- | --- | --- | --- | --- | --- | --- | --- | --- | --- |
| PIH1 | 310346707 | 126800326 (40.9%) | 121807797 (39.2%) | 4992529 (1.6%) | 64732597 (20.9%) | 59583256 (19.2%) | 5149341 (1.7%) | 37953949 (12.2%) | 34438520 (11.1%) | 3515429 (1.1%) |
| PIH2 | 334744537 | 104739437 (31.3%) | 100943871 (30.2%) | 3795566 (1.1%) | 90550433 (27.1%) | 84407406 (25.2%) | 6143027 (1.8%) | 38046038 (11.4%) | 34843110 (10.4%) | 3202928 (1.0%) |
| PIH3 | 290454314 | 112526632 (38.7%) | 107470654 (37.0%) | 5055978 (1.7%) | 63801134 (22.0%) | 58020736 (20.0%) | 5780398 (2.0%) | 31846024 (11.0%) | 28298253 (9.7%) | 3547771 (1.2%) |
| PIH4 | 279158823 | 119025809 (42.6%) | 114497964 (41.0%) | 4527845 (1.6%) | 53629842 (19.2%) | 48719851 (17.5%) | 4909991 (1.8%) | 35699441 (12.8%) | 32033663 (11.5%) | 3665778 (1.3%) |
| NC2 | 307855917 | 37017249 (12.0%) | 35564855 (11.6%) | 1452394 (0.5%) | 115331559 (37.5%) | 108482738 (35.2%) | 6848821 (2.2%) | 31628561 (10.3%) | 29399646 (9.5%) | 2228915 (0.7%) |
| NC3 | 318333874 | 39871451 (12.5%) | 38494498 (12.1%) | 1376953 (0.4%) | 76739925 (24.1%) | 72614707 (22.8%) | 4125218 (1.3%) | 18541058 (5.8%) | 17080689 (5.4%) | 1460369 (0.5%) |
| NC4 | 392197177 | 100615883 (25.7%) | 97060644 (24.7%) | 3555239 (0.9%) | 77995021 (19.9%) | 73001295 (18.6%) | 4993726 (1.3%) | 2932450 (8.4%) | 30046020 (7.7%) | 2886430 (0.7%) |
