# Supplementary material for: Pregnancy induced hypertension and umbilical cord blood DNA methylation in newborns: an epigenome-wide DNA methylation study
Source: BMC Pregnancy Childbirth. 2024 Jun 17;24:433. doi: 10.1186/s12884-024-06623-8 (PMC11181590; doi:10.1186/s12884-024-06623-8)
Supplement: Supplementary file 3 — Supplementary Material 3. [file 12884_2024_6623_MOESM3_ESM.docx]

Table S2. The result of deduplication of the sorted bam file by the sambamba software.

| Sample | Sorted end pairs | Single ends | Unmatehed pairs | Duplicates | Batch |
| --- | --- | --- | --- | --- | --- |
| PIH1 | 116300759 | 77147924 | 516734 | 86444559 | Batch1 |
| PIH2 | 125469936 | 86697498 | 561497 | 92985938 | Batch1 |
| PIH3 | 109153611 | 102831036 | 522881 | 92894198 | Batch1 |
| PIH4 | 110953484 | 79353329 | 516233 | 75980907 | Batch1 |
| NC2 | 50353803 | 108304488 | 333378 | 73619360 | Batch1 |
| NC3 | 43536230 | 79611932 | 163842 | 30982555 | Batch2 |
| NC4 | 100767413 | 95633777 | 283966 | 58009596 | Batch2 |
