# Supplementary material for: Pregnancy induced hypertension and umbilical cord blood DNA methylation in newborns: an epigenome-wide DNA methylation study
Source: BMC Pregnancy Childbirth. 2024 Jun 17;24:433. doi: 10.1186/s12884-024-06623-8 (PMC11181590; doi:10.1186/s12884-024-06623-8)
Supplement: Supplementary file 4 — Supplementary Material 4. [file 12884_2024_6623_MOESM4_ESM.docx]

**Table 3 Gene list for Top 20 DMR**

|  | **Position** | **strand** | **pvalue** | **meth.diff** | **No. of CPGs** | **GeneSymbol** | **location** |
| --- | --- | --- | --- | --- | --- | --- | --- |
| hyperDMR | Chr5:77828001-77829000 | * | 2.17476E-06 | 44.75890985 | 2 | TBCA | intergenic |
|  | Chr6:97834001-97835000 | * | 7.47417E-08 | 43.15611595 | 39 | AL589740.1 | intergenic |
|  | Chr15:81791001-81792000 | * | 5.87876E-06 | 37.73204197 | 5 | AC104041.1 | intergenic |
|  | Chr1:187153001-187154000 | * | 9.56215E-06 | 36.33879781 | 7 | ERVMER61-1 | ncRNA_intronic |
|  | Chr4:129074001-129075000 | * | 2.18808E-05 | 35.19151847 | 8 | SCLT1 | intronic |
|  | Chr6:145552001-145553000 | * | 2.35863E-05 | 34.53654189 | 3 | EPM2A | intergenic |
|  | Chr1:70756001-70757000 | * | 4.36477E-09 | 34.29487179 | 2 | LINC01788 | ncRNA_intronic |
|  | Chr4:161677001-161678000 | * | 9.9993E-06 | 33.5026412 | 4 | FSTL5 | intronic |
|  | Chr7:33125001-33126000 | * | 3.63404E-07 | 32.93144208 | 2 | BBS9 | intergenic |
|  | Chr1:242251001-242252000 | * | 0.000194192 | 30.07407407 | 8 | PLD5 | intronic |
|  | Chr4:112071001-112072000 | * | 0.000244695 | 30 | 4 | AC004704.1 | intergenic |
|  | Chr6:34956001-34957000 | * | 8.34629E-05 | 28.71386678 | 6 | ANKS1A | intronic |
|  | Chr12:10862001-10863000 | * | 7.64197E-06 | 28.34224599 | 2 | PRH1 | ncRNA_intronic |
|  | Chr8:3498001-3499000 | * | 0.00016233 | 28.22453485 | 12 | CSMD1 | intronic |
|  | Chr1:113569001-113570000 | * | 6.87553E-06 | 26.88137755 | 8 | MAGI3 | intronic |
|  | Chr2:80703001-80704000 | * | 0.00018675 | 25.49232159 | 4 | AC012355.1 | intergenic |
|  | Chr9:102616001-102617000 | * | 1.38183E-05 | 25.47688921 | 4 | LINC00587 | ncRNA_intronic |
|  | Chr14:100027001-100028000 | * | 0.000568971 | 25.29411765 | 6 | EVL | intronic |
|  | Chr18:46741001-46742000 | * | 0.000284912 | 25.10504202 | 7 | ST8SIA5 | intronic |
|  | Chr2:58196001-58197000 | * | 0.000451331 | 24.68102073 | 11 | FANCL | intronic |
| hypoDMR | Chr14:24641001-24642000 | * | 1.10E-11 | -56.22425933 | 2 | AL136018.1 | intergenic |
|  | Chr15:87335001-87336000 | * | 1.38E-15 | -44.6512623 | 3 | AC020687.1 | intergenic |
|  | Chr20:42504001-42505000 | * | 6.94E-06 | -39.90729035 | 17 | PTPRT | intronic |
|  | Chr2:141759001-141760000 | * | 1.15E-06 | -38.31378299 | 5 | LRP1B | intronic |
|  | Chr22:47824001-47825000 | * | 1.63E-04 | -32.09494324 | 34 | AL117329.1 | ncRNA_intronic |
|  | Chr1:96780001-96781000 | * | 1.05E-10 | -31.86313291 | 9 | PTBP2 | intronic |
|  | Chr8:32353001-32354000 | * | 1.8725E-05 | -30.62801932 | 10 | NRG1 | intronic |
|  | Chr6:129133001-129134000 | * | 2.51E-03 | -30.58312655 | 6 | LAMA2 | intronic |
|  | Chr5:170329001-170330000 | * | 9.14E-07 | -30.38488701 | 2 | LINC01366 | intergenic |
|  | Chr14:39638001-39639000 | * | 8.74371E-07 | -29.16575672 | 5 | AL049828.1 | intergenic |
|  | Chr5:120247001-120248000 | * | 1.60E-03 | -28.88888889 | 4 | AC008574.1 | intergenic |
|  | Chr4:88961001-88962000 | * | 3.97E-05 | -28.87172887 | 6 | FAM13A | intronic |
|  | Chr10:49100001-49101000 | * | 1.12E-03 | -28.57142857 | 4 | VSTM4 | intronic |
|  | Chr12:55511001-55512000 | * | 7.53E-04 | -28.33843017 | 3 | AC122685.1 | intergenic |
|  | Chr6:76865001-76866000 | * | 2.02E-07 | -24.94057921 | 3 | AL355612.1 | intergenic |
|  | Chr9:13525001-13526000 | * | 2.93E-03 | -23.92156863 | 2 | AL583785.1 | intergenic |
|  | Chr2:144838001-144839000 | * | 1.01E-06 | -23.78498816 | 357 | TEX41 | ncRNA_intronic |
|  | Chr3:191469001-191470000 | * | 6.14E-03 | -23.61673414 | 50 | AC073365.1 | ncRNA_intronic |
|  | Chr2:103942001-103943000 | * | 1.02E-03 | -23.3409611 | 4 | LINC01965 | intergenic |
|  | Chr5:64246001-64247000 | * | 5.08E-03 | -22.71352986 | 4 | RNF180 | intronic |
